# Generation and Characterization of a Diabody Targeting the α_v_β_6_ Integrin

**DOI:** 10.1371/journal.pone.0073260

**Published:** 2013-09-04

**Authors:** Heide Kogelberg, Enrique Miranda, Jerome Burnet, David Ellison, Berend Tolner, Julie Foster, Carmen Picón, Gareth J. Thomas, Tim Meyer, John F. Marshall, Stephen J. Mather, Kerry Chester

**Affiliations:** 1 UCL Cancer Institute, University College London, London, United Kingdom; 2 Centre for Molecular Oncology, Barts Cancer Institute, Queen Mary University of London, John Vane Science Centre, London, United Kingdom; 3 Cancer Sciences Unit, University of Southampton, Southampton, United Kingdom; 4 Centre for Tumour Biology, Barts Cancer Institute, Queen Mary University of London, John Vane Science Centre, London, United Kingdom; National Cancer Institute, NIH, United States of America

## Abstract

The α_v_β_6_ integrin is up-regulated in cancer and wound healing but it is not generally expressed in healthy adult tissue. There is increasing evidence that it has a role in cancer progression and will be a useful target for antibody-directed cancer therapies. We report a novel recombinant diabody antibody fragment that targets specifically α_v_β_6_ and blocks its function. The diabody was engineered with a C-terminal hexahistidine tag (His tag), expressed in *Pichia pastoris* and purified by IMAC. Surface plasmon resonance (SPR) analysis of the purified diabody showed affinity in the nanomolar range. Pre-treatment of α_v_β_6_-expressing cells with the diabody resulted in a reduction of cell migration and adhesion to LAP, demonstrating biological function-blocking activity. After radio-labeling, using the His-tag for site-specific attachment of ^99m^Tc, the diabody retained affinity and targeted specifically to α_v_β_6_-expressing tumors in mice bearing isogenic α_v_β_6_ +/− xenografts. Furthermore, the diabody was specifically internalized into α_v_β_6_-expressing cells, indicating warhead targeting potential. Our results indicate that the new α_v_β_6_ diabody has a range of potential applications in imaging, function blocking or targeted delivery/internalization of therapeutic agents.

## Introduction

The α_v_β_6_ integrin is an epithelial restricted trans-membrane protein that has emerged as a promising target for antibody-directed therapies. It is up-regulated in many tumor types including pancreatic ductal adenocarcinoma, head and neck squamous cell carcinoma, ovarian cancer, colon cancer, cholangiocarcinoma and cervical cancer [1], [2], [3], [4], [5]. During embryogenesis and wound healing α_v_β_6_ promotes binding to extracellular matrix proteins (fibronectin, vitronectin and tenascin) facilitating cell migration [6] and activates TGFβ_1_ via binding to the latency associated peptide (LAP) of the TGFβ complex [7]. In cancer, α_v_β_6_ has been shown to modulate invasion, inhibit apoptosis and regulate expression of matrix metalloproteases (MMPs) [8]. Importantly for a cancer target, α_v_β_6_ is only found at very low levels in normal tissue; its expression has been reported to regulate wound healing [6] and activation of TGFβ_1_ in response to injury and inflammation in the lungs [9].

The various roles of α_v_β_6_ in cancer have not yet been fully elucidated, although it has been shown to be a contributing factor in tumor progression [4], [10] and has been associated with enhanced tumorigenic properties in colon carcinoma facilitating liver metastasis [4], [11], and reducing survival times in gastric carcinoma [10]. Expression of α_v_β_6_ has been reported during epithelial-mesenchymal transition (EMT) and it is thought to have a role in sustaining the EMT process [12], [13]. Interestingly, high levels of α_v_β_6_ are found in the context of K-Ras dependency in lung and pancreatic cancer cell lines [14]. Depletion of the ITGB6 gene had a clear growth inhibitory effect on these cells [15], indicating that α_v_β_6_ may be a tractable target in K-Ras mutant cancers. Targeting this integrin has shown tumor growth inhibition *in vivo* due to blockade of α_v_β_6_-dependent activation of the TGFβ pathway [16].

Antibodies reactive specifically with α_v_β_6_ could have diagnostic and therapeutic utility, particularly if they have function blocking activity. Towards this, we previously engineered murine and humanized single chain Fv antibody fragments (scFvs) reactive with α_v_β_6_ [17]. The unique α_v_β_6_ specificity was gained by an insertion into the CDR3 loop of the variable heavy-chain (VH) domain of an existing scFv scaffold. Here we describe the development of an anti-α_v_β_6_ scFv into a stable *in vivo* targeting agent in diabody format. Diabodies are non-covalently associated bivalent molecules, created from scFvs by shortening the polypeptide linker between the VH and VL domains [18]. Their bivalent nature is advantageous for targeting [19], [20], [21] and they provide a flexible platform for development of targeted therapeutics, particularly since their pharmacokinetics are readily modified by attachment of polyethylene glycol [22]. We show that the anti-α_v_β_6_ diabody blocks α_v_β_6_-mediated biological functions. Moreover, the ^99m^Tc-labeled diabody targeted specifically to α_v_β_6_+ve tumors *in vivo* within 2 hours of administration.

## Materials and Methods

### Cell lines

A375Pβ6 is a α_v_β_6_-positive human cell line, generated through retroviral transduction of the melanoma cell line A375P with human β_6_ cDNA and a puromycin-resistance gene as described previously [23]. The control cell line, A375Ppuro was transduced with the puromycin-resistance gene alone [23]. Both cell lines express several other RGD-binding integrins at equivalent levels, namely α_5_β_1,_ α_v_β_3_, α_v_β_5_, α_v_β_8_ [17]. Capan-1 (α_v_β_6_-positive human pancreatic cell line) was obtained from ATCC (HTB-79). All cell lines were maintained in Dulbecco's modified Eagle's medium (DMEM) (PAA Laboratories, UK) supplemented with 2 mM L-glutamine (PAA Laboratories, UK) and 10% foetal calf serum (Labtech International, Ringmer, UK).

### Production of B6.3 and shMFE23 diabody proteins

The diabody was generated from the B6.3 scFv vH and vL domains [17] by synthesizing the scFv gene with a G_4_S linker. The synthesized gene was obtained from Genescript (Piscataway, NJ, USA) and cloned into the pPICZαBHis vector (Invitrogen) as described previously [17]. The resulting plasmid was linearized with *Pme*I, transformed into electrocompetent *P. pastoris* X33 cells (Invitrogen) and transformants grown on YPDS and Zeocin (100 μg/ml; Invitrogen) plates. Positive clones were selected and screened for methanol-induced protein expression according to the manufacturer's recommendations. Clones with the highest B6.3 diabody expression were used for protein production by fermentation with initial purification using expanded-bed adsorption IMAC as previously described [24], [25]. The B6.3 diabody was harvested 4 h post induction of protein expression. Final purification was performed by size-exclusion chromatography on a Superdex 75 (GE Healthcare) column (500-ml bed volume) equilibrated with phosphate-buffered saline (PBS), pH 7.4. For ^99m^Tc labeling experiments the diabody was further concentrated to 7.3 mg/ml by application to a 1ml Ni^2+^-charged HiTrap IMAC SP FF column (GE Healthcare) according to the manufacturer's instructions. Purified protein was analyzed by SDS-PAGE using Tris-glycine gels (16%; Invitrogen) and stained with Coomassie brilliant blue R250 (Sigma). The shMFE23 diabody was produced following the same protocol as for B6.3 diabody production, using the shMFE23 scFv [26] vH and vL domains as template. The purified protein gave a single peak by size exclusion chromatography (data not shown) indistinguishable from that obtained with the B6.3 diabody.

### Affinity of α_v_β_6_ binding to B6.3 diabody by Surface Plasmon Resonance

Affinity of purified B6.3 diabody for α_v_β_6_ was measured by surface plasmon resonance (SPR) using a Biacore T100. The diabody was immobilized on a Research Grade CM5 chip using an amine coupling kit (BIAcore, GE Healthcare). Recombinant α_v_β_6_ protein (R&D Systems) was flown over the immobilized B6.3 diabody in HBS-P buffer (10 mM HEPES, 150 mM NaCl, 0.05% v/v Surfactant P20, pH 7.4, with addition of 2 mM Ca^2+^ and 2 mM Mg^2+^ ions) at 30 μL/min at 25°C. Association and dissociation phases occurred over 300 s. Kinetics of binding was calculated from data at 400 nM, 200 nM, 100 nM, 50 nM, 25 nM, 12.5 nM, 6.25 nM and 3.125 nM using the BIAevaluation program. The surface was regenerated with 10 mM Glycine-HCl, pH 2.5. The affinity constant (KD) was obtained by simultaneously fitting the association and dissociation phases of the sensogram from the analyte concentration series using the 1∶1 Langmuir model (BIAevaluate software).

### Flow cytometric analysis of B6.3 diabody binding to α_v_β_6_-expressing cells

A375Pβ6 and A375Ppuro cells were trypsinized, re-suspended in DMEM supplemented with 0.1% (v/v) BSA and 0.1% (w/v) sodium azide (DMEM0.1/0.1) to approximately 5×10^6^ cells/ml and incubated with various concentrations of B6.3 diabody. Bound diabody was detected with mouse Tetra-His antibody (1 μg/ 100 μl, Qiagen) and R-PE-conjugated goat anti-mouse IgG (BD Pharmingen, 1 μg/100μl). Detection antibodies were incubated in DMEM0.1/0.1 for 45 min at 4°C; all incubations were followed by washing with DMEM0.1/0.1. Cells were fixed with IntraStain kit (DakoCytomation, Glostrup, Denmark) and analyzed by flow cytometry using a CyAn ADP High-Performance Flow Cytometer (Becton Dickinson). For binding inhibition studies, A375Pβ6 cells were incubated with mouse anti-α_v_β_6_ (10D5, Chemicon International) at various concentrations for 15vmin followed by incubation with 100 ng (18.04 nM) of diabody for 30 min. After washing, bound diabody was detected with rabbit anti-hexahistidine IgG (GenScript) at 1 μg/100 μl, followed by R-PE-conjugated goat anti-rabbit IgG (1 μg/100 µl, Invitrogen). All incubation and washing steps were in DMEM0.1/0.1 at 4°C. Cells were fixed and analysed as described above.

### ^99m^Tc labeling of B6.3 diabody

Sodium [^99m^Tc] pertechnetate was obtained from a ^99^Mo/^99m^Tc generator (GE Healthcare, Amersham UK) and converted to [^99m^Tc(CO)_3_(H_2_O)_3_]^+^using an IsoLink^TM^ kit (generously provided by Covidien, Petten, The Netherlands) according to the manufacturer's instructions. B6.3 diabody was labeled at the C-terminal hexahistidine tag with ^99m^Tc by incubating with 750MBq of [^99m^Tc(CO)_3_(H_2_O)_3_]^+^ in a total volume of 574 µl at 37°C for 2 h. The labeled protein was separated from the non-incorporated radionuclide by desalting (NAP-10 column, GE Healthcare). Integrity of the radio-labeled protein as a dimer was verified by size-exclusion HPLC on a Biosep-SEC-S 2000 column eluted with 0.1 M phosphate buffer pH 7 at a flow rate of 0.5 ml/min.

### Cell Saturation Binding Assay

The immunoreactivity and affinity of ^99m^Tc-labeled B6.3 diabody to α_v_β_6_ was analyzed by a saturation-binding assay using A375Pβ6 cells. Six duplicate test samples containing increasing amounts of ^99m^Tc-labeled B6.3 diabody and approximately 6.5×10^5^ A375Pβ6 cells per experiment were incubated in a total volume of 1ml of DMEM with 0.1% (v/v) BSA (DMEM0.1) at 4°C for 3 h. Supernatant was removed by centrifugation and cells were washed once with DMEM0.1. An identical series of tubes were prepared in which non-specific binding was determined by addition of 25 µg unlabeled diabody to each tube. Non-specific binding was subtracted from total binding to obtain specific binding. Affinity constant (KD) and maximal number of α_v_β_6_ binding sites (Bmax) were determined by non-linear regression analysis using Graphpad prism software.

### Immunofluorescence microscopy of internalization of B6.3 diabody into α_v_β_6_-expressing cells

A375Pβ6 cells were seeded on to glass cover slips at 2×10^5^ cells/well and incubated for 48 h at 37°C. Cells were then washed with DMEM0.1, incubated with 5 µg/ml of B6.3 diabody in 1%BSA/DMEM (DMEM1) for 1 h at 4°C and subsequently washed and incubated in 10% (v/v) FBS/DMEM at 37°C for various time points. After incubation, cells were washed twice with Tris-Cl, pH 7.5, containing 2 mM Ca^2+^ and 1 mM Mg^2+^ (Tris/M), followed by fixation in 4% paraformaldehyde/Tris/M for 20 min on ice. After washing with PBS, cells were incubated with 10 mM ammonium chloride/PBS for 10 min at room temperature and permeabilized with ice-cold methanol. Finally, cells were blocked with 1% (w/v) BSA/PBS for 30 min at room temperature and stained with 1 μg/ml of rabbit anti-human IgG (Jackson Immuno Research, Suffolk, UK) in 1% (w/v) BSA/PBS followed by Alexa Fluor 546®-labeled goat anti-rabbit IgG (1∶500) (Invitrogen), containing Hoechst trihydrochloride (1∶5000) (Invitrogen) in 1% (w/v) BSA/PBS, each for 1 h at 4°C. Cover slips were mounted on slides using ProLong Gold antifade (Invitrogen) and examined using Perkin Elmer Spinning Disc Confocal microscope and Volocity^TM^ Visualisation Software.

### Adhesion assays

Ninety-six-well plates were coated with 100 µl of fibronectin (R&D Systems) at 25 µg/ml or LAP (R&D Systems) at 0.5 µg/ml for 1 h at 37°C. After coating, plates were washed with PBS and blocked with 1%BSA/PBS at 37°C for 1 h. For blocking experiments, cells were treated with 50 µg/ml B6.3 diabody for 1 h at 4°C in DMEM0.1 and seeded at 5×10^4^ cells/well. After incubation at 37°C for 1 h, plates were extensively washed with PBS to remove non-attached cells and 100 µl of a dilution 1∶10 of Prestoblue^©^ (Invitrogen) was added to each well. Fluorescence signal was measured after incubation at 37°C for 4 h using a Multimode Varioskan plate reader (Thermo Scientific). Results were expressed as percentage of attachment relative to untreated cells with statistical significance analyzed by Student's unpaired t-test.

### Migration assays

Cell migration was analysed using Transwell assays (Corning, NY, USA) with polycarbonate filters (8 µm pore size). Membrane undersurface was coated with fibronectin (R&D Systems) at 25 µg/ml or LAP (R&D Systems) at 0.5 µg/ml for 1h at 37°C and blocked with DMEM0.1 for 1 h at 37°C. Cells were treated as described for adhesion assays and seeded in the upper chamber at 1×10^5^ cells/chamber in 100 µl. The lower chamber was filled with 600 µl DMEM0.1. Plates were then incubated for 20 h at 37°C and cells in the upper chamber were carefully removed using a cotton swap. Migrated cells were fixed with 4% paraformaldehyde, stained with Hoechst trihydrochloride (1∶5000) (Invitrogen) for 10 min and counted using a Zeiss AxioImager A1 fluorescence microscope with AxioVision software. Results were expressed as percentage of migrated compared to untreated cells with statistical significance analyzed by Student's unpaired t-test.

### Immunocytofluorescence for Smad2/3 localization

Capan-1 cells were seeded in cover slips in 10% (v/v) FBS/DMEM and allowed to grow to 70% confluency. Cells were then washed twice in PBS, starved for 24 h in serum-free DMEM and treated with 50 µg/ml B6.3 diabody for 1 h at 4°C in DMEM0.1. After incubation with the diabody cells were washed twice with PBS and treated with DMEM0.1, latent TGFβ_1_ (Cell Signaling Technology) at 50 ng/ml or TGFβ_1_ (R&D Systems) at 10 ng/ml for 30 min at 37°C. After extensive washing with PBS, 4% formaldehyde was used to fix the cells (15 min at 4°C). Cells were then permeabilized with 0.3% Triton-X 100 (Sigma) in PBS and cover slips were blocked for 1 h with 5% goat serum before overnight incubation with rabbit anti-Smad2/3 antibody (Cell Signaling Technology) at 4°C. The following day, primary antibody was detected with Alexa Fluor 488®-labeled goat anti-rabbit IgG (Invitrogen) containing Hoechst trihydrochloride (Invitrogen). Cover slips were mounted on slides using ProLong Gold antifade (Invitrogen) and examined using a Zeiss AxioImager A1 fluorescence microscope with AxioVision software.

### *In vivo* studies

All experiments were conducted with previous approval from the UK Home Office, under PPL 70/6677. Female SCID mice were injected subcutaneously (s.c.) with 4×10^6^ A375Pβ6 cells in one flank, and 4×10^6^ A375Ppuro cells in the contralateral flank, in 150 µl serum-free DMEM. Once tumors reached a diameter of around 5 mm, approximately 11 µg (30MBq) per mouse of ^99m^Tc-labeled B6.3 diabody in 200 µl PBS was injected intravenously (i.v.). Mice were anaesthetised with isoflurane and imaged 2 h, 5 h and 24 h after injection using a Nano-SPECT/CT scanner (Bioscan, Washington, DC, USA). SPECT images were analysed using *in vivo* Scope software (Bioscan). Mice were sacrificed 24 h after injection of diabody; tissues were excised and radioactivity measured on a gamma counter (LKB Compugamma, Victoria, Australia) alongside standards prepared from the injectate. Uptake of radioactivity in individual tissues was expressed as a percentage of the injected radioactive dose per gram (%ID/g).

## Results

### Expression and characterisation of B6.3 diabody

**Figure 1:**
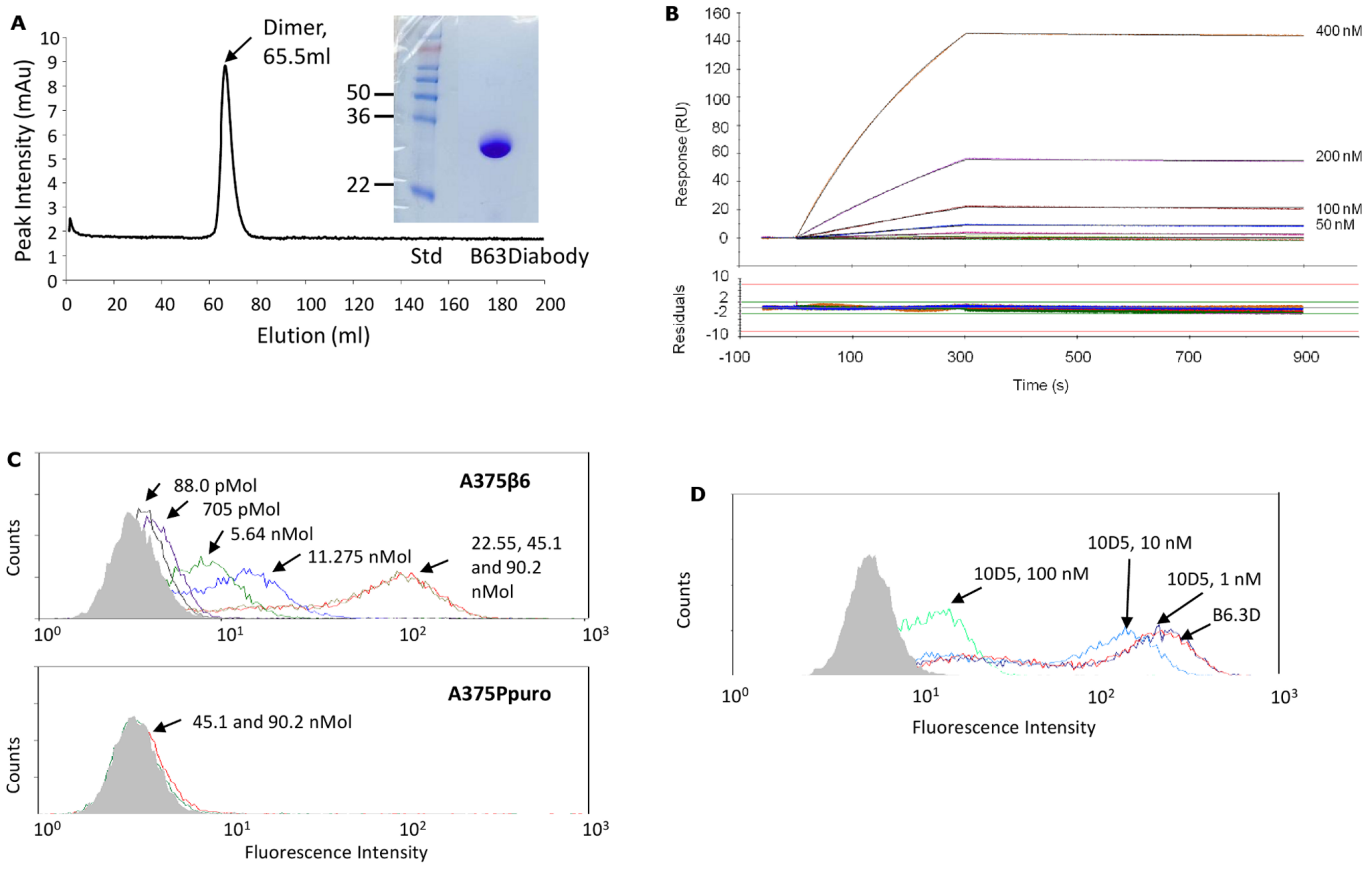
Production of B6.3 diabody and analysis of its specific interaction with α_v_β_6_. A) Size-exclusion chromatographic profile (Superdex 75, 125 ml) of B6.3 diabody after fermentation, expanded-bed adsorption IMAC, Superdex 75 (500 ml), 1 ml Ni^2+^-charged Hi-Trap IMAC, freezing and de-frosting. B6.3 diabody eluted from the column as a dimer that separated in monomeric form under reducing conditions by SDS-PAGE, consistent with non-covalent association of monomers in a diabody structure. B) Sensogram of real-time binding and dissociation of B6.3 diabody to α_v_β_6_. B6.3 diabody was immobilized on a BIAcore CM5 sensor chip and α_v_β_6_ protein was flown across at 400, 200, 100, 50, 25, 12.5, 6.25 and 3.125 nM. The affinity constant (KD) for the interaction was 2.8×10^−9^ M, with on-rate of 8,107±7.3 M^−1^s^−1^ and off-rate of 2.3×10^−5^±1.4×10^−7^ s^−1^. C) Flow cytometry analysis of B6.3 diabody binding to α_v_β_6_-expressing A375Pβ6 cells in a concentration-dependent manner (a) but not to A375Ppuro cells (b), which do not express this integrin. Cells were incubated with B6.3 diabody, at the indicated concentrations and binding was detected with mouse anti-tetra-histidine IgG followed by R-PE-labeled goat anti-mouse IgG. B6.3 diabody was not added to omission control (shown in solid grey). D) Inhibition of B6.3 diabody binding after incubation with the anti-α_v_β_6_ antibody 10D5 shown by flow cytometry. Cells were incubated with 100 ng B6.3 diabody with or without prior incubation with 10D5 at the indicated concentrations. Binding of B6.3 diabody was detected with rabbit anti-hexahistidine IgG followed by R-PE-labeled goat anti-rabbit IgG. In the omission control experiment (shown in solid grey) cells were not incubated with B6.3 diabody and 10D5.

B6.3 diabody was generated as soluble protein by fermentation in *P. pastoris* giving a yield of 175 mg/L. The diabody was purified from the bioreactor broth using expanded bed IMAC, exploiting the engineered hexahistidine tag, and concentrated to 2.27 mg/ml. There was no evidence of aggregation when the product was tested by size-exclusion chromatography; diabody eluted as a single peak of >44 kDa, consistent with its calculated MW of 55,322Da (Fig. 1A). The protein was essentially pure as shown by SDS-PAGE and was revealed as a monomer under denaturating conditions (Fig. 1A), consistent with diabody formation by non-covalent association. We next analysed the binding affinity of the purified B6.3 diabody to α_v_β_6_. SPR showed that α_v_β_6_ bound to B6.3 diabody in a concentration-dependent manner (Fig. 1B) and subsequently remained associated. Fitting of the data to a Langmuir 1∶1 model gave an affinity constant (KD) value of 2.78−10^−9^M and kinetic rate constants of ka  = 8.1×103±7.3 s^−1^M^−1^ and kd  = 2.3×10^−5^±1.4×10^−7^s^−1^.

### Interactions with α_v_β_6_-expressing cells

Specificity of purified B6.3 diabody for α_v_β_6_ on tumor cells was assessed by flow cytometry using the α_v_β_6_-expressing cell line, A375Pβ6, and corresponding α_v_β_6_-negative A375Ppuro cells. The results showed that B6.3 diabody bound to the α_v_β_6_-expressing cells in a concentration-dependent manner (Fig. 1C) but did not bind to the α_v_β_6_-negative cells when tested at the two highest concentrations (Fig. 1C). The shift in fluorescence intensity observed for binding to A375Pβ6 cells were similar at 22.6, 45.1 and 90.2 nM, indicating that antigen saturation was reached at these concentrations. To further verify the specificity of the B6.3 diabody to α_v_β_6_, cells were pre-incubated with an anti-α_v_β_6_ antibody, 10D5. This resulted in inhibition of B6.3 diabody binding when 10D5 was used at 10 and 100 nM (Fig. 1D).

**Figure 2:**
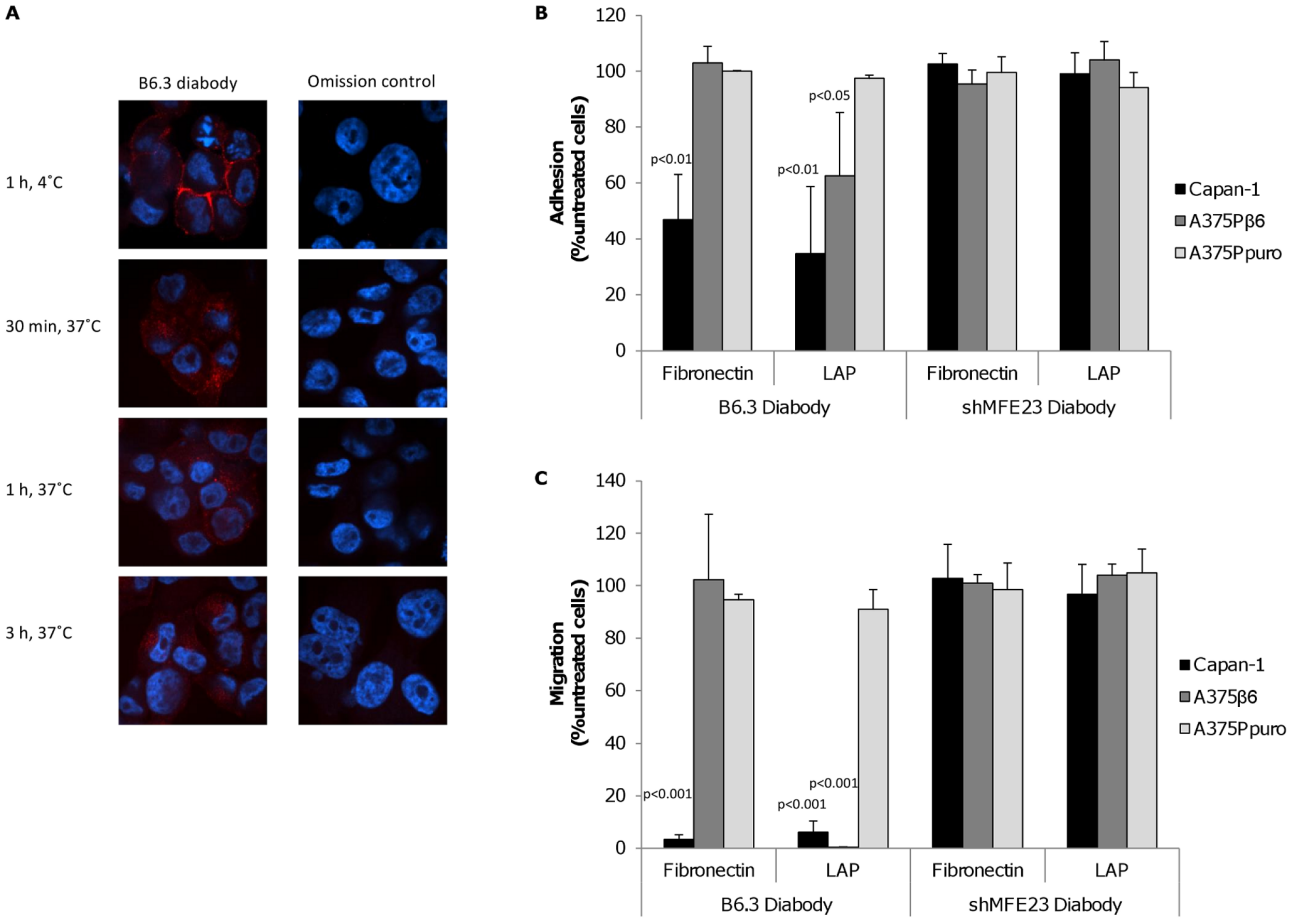
Treatment of α_v_β_6_-expressing cells with B6.3 diabody resulted in diabody internalization and blockade of integrin functions. A) Localization of B6.3 diabody in A375Pβ6 cells by confocal microscopy. B6.3 diabody detection showed membrane pattern of staining at 4°C and internalized when cells were incubated at 37°C for 30 min, 1 h and 3 h. B6.3 diabody was detected using rabbit anti-human IgG followed by Alexa Fluor 546®-labeled goat anti-rabbit IgG (red). Cells were also counterstained with Hoechst 33245 (blue). B) Treatment of α_v_β_6_-expressing cells blocked adhesion to LAP-coated plates (A375Pβ6 and Capan-1 cells) and/or fibronectin-coated plates (Capan-1 cells). Cells were incubated with B6.3 or shMFE23 diabody at 4°C for 1 h and allowed to attach to coated plates for 1 h at 37°C. Treatment with the anti-CEA shMFE23 diabody had no effect on the cell lines used. C) B6.3 diabody treatment inhibited migration towards LAP and fibronectin. As observed in adhesion assays, the diabody inhibited migration of A375Pβ6 cells to LAP and migration of Capan-1 cells to fibronectin and LAP, while targeting CEA had no effect on the cells tested.

Next we tested whether the diabody would internalize specifically into the α_v_β_6_-expressing cells, as previously reported for other ligand-mimic antibodies targeting this integrin [27]. Cells were treated with B6.3 diabody at 4°C for 1 h to allow binding to the outer cell membrane. Then, temperature was increased to 37°C for different length of time to allow internalization and the resulting cellular distribution of the diabody was revealed after fixation and permeabilization by fluorescence staining. Results of these experiments showed that, upon binding at 4°C, B6.3 diabody was localized at the cell surface (Fig. 2A). When the temperature was raised to 37°C surface staining disappeared and the diabody was found inside the cells after 30 min, 1 h and 3 h of incubation (Fig. 2A).

### B6.3 diabody-mediated blockade of α_v_β_6_ biological functions

The α_v_β_6_ integrin is known to have a role in the promotion of cell migration based on interaction with components of the extracellular matrix [6], [11], [16]. Since the B6.3 diabody contained an RGD motif and internalized as a ligand-mimic antibody, we investigated whether the diabody would exhibit biological effects associated with integrin blockade. First we tested the ability of the diabody to inhibit adhesion and migration of α_v_β_6_-positive or negative cells to the α_v_β_6_ ligands, LAP and fibronectin. In addition to the stably-transfected A375Pβ6 cells, we included the naturally α_v_β_6_-expressing pancreatic cancer cell line Capan-1. Treatment with B6.3 diabody at 50 µg/ml resulted in a reduction in adhesion of A375Pβ6 and Capan-1 cells to LAP-coated plates (Fig. 2B). In addition, we observed a decrease in the number of Capan-1 cells attached to fibronectin-coated plates. The shMFE23 diabody targeting the carcinoembryonic antigen (CEA) was used as control; pre-treatment with this diabody had no effect on the CEA-positive Capan-1 cells or the melanoma cell lines, negative for CEA [17]. The results from Transwell migration assays were more marked as the B6.3 diabody induced an almost complete inhibition of migration towards LAP in both A375Pβ6 and Capan-1 cells (Fig. 2C). In addition, the migration of Capan-1 cells to fibronectin was almost completely inhibited by the diabody, indicating that α_v_β_6_ is the major fibronectin-binding integrin on Capan-1. In contrast, B6.3 diabody did not block A375Pβ6 cells adhering to or migrating on fibronectin (Fig 2C), consistent with our previous data that showed both A375Pβ6 and A375Ppuro express two other fibronectin-binding integrins α_v_β_3_ and α_5_β_1_ [17]. No effect on adhesion or migration was observed in the α_v_β_6_-negative cell line A375Ppuro or after addition of an irrelevant diabody, indicating that the B6.3-dependent inhibition was α_v_β_6_-specific.

**Figure 3:**
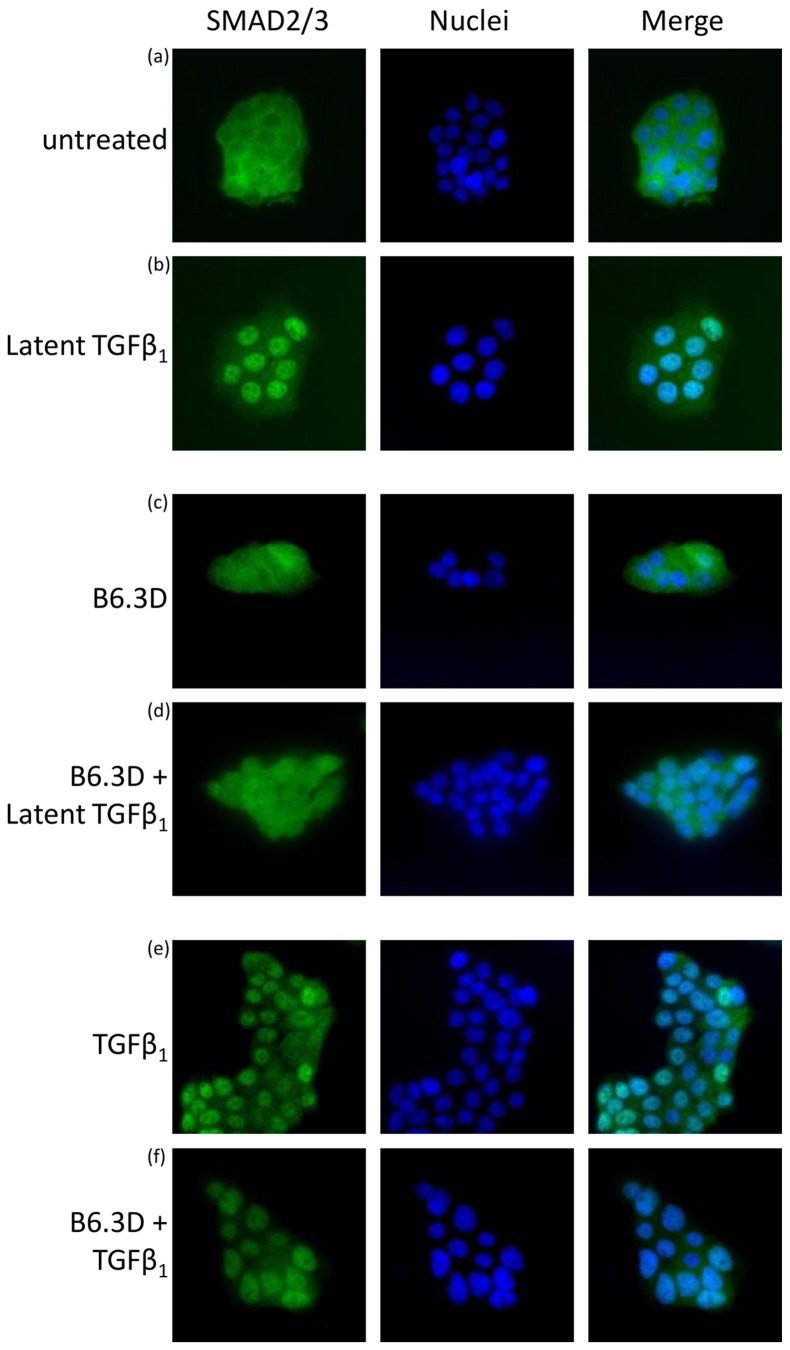
B6.3 diabody inhibited LAP-mediated Smad2/3 translocation to the nucleus in Capan-1 cells. Cells were incubated at 4°C in the presence of B6.3 diabody and then treated with LAP or TGFβ_1_ (30 min, 37°C); Smad2/3 localization was assessed by confocal microscopy (40X) using rabbit anti-Smad2/3 followed by Alexa Fluor 488®-labeled goat anti-rabbit IgG (green); smad2/3 was found in the cytoplasm of starved Capan-1 cells (a) and after treatment with B6.3 diabody (c). Smad2/3 was present in the nuclei in response to treatment with latent TGFβ_1_ (b), a translocation that was inhibited by pre-treatment B6.3 diabody (d). TGFβ_1_ was used as a positive control (e.f).

The α_v_β_6_ integrin is known to activate TGFβ_1_ upon interaction with its latent form (LAP-TGFβ_1_ complex), resulting in TGFβ-induced smad2/3 phosphorylation and subsequent translocation of smad2/3 to the nucleus [7]. We hypothesized that binding of B6.3 diabody to α_v_β_6_ would inhibit its interaction with latent TGFβ_1_ and downstream Smad2/3 translocation. We showed that smad2/3 localized in the cytoplasm in serum-starved Capan-1 cells (Fig. 3(a)). Incubation with B6.3 diabody had no effect on smad2/3 localization (Fig. 3(c)), while treatment with latent TGFβ_1_ resulted in nuclear translocation of Smad2/3 (Fig. 3(b)). We next tested the localization of smad2/3 after incubation with the B6.3 diabody. Our data showed that B6.3 diabody inhibited Smad2/3 nuclear translocation mediated by latent TGFβ_1_ (Fig. 3(d)), indicating that the diabody blocked smad2/3 activation. Active TGFβ_1_ showed Smad2/3 translocation in B6.3-treated cells (Fig. 3(f)), since the active form does not require prior integrin interactions.

### Labeling efficiency of B6.3 diabody

**Figure 4:**
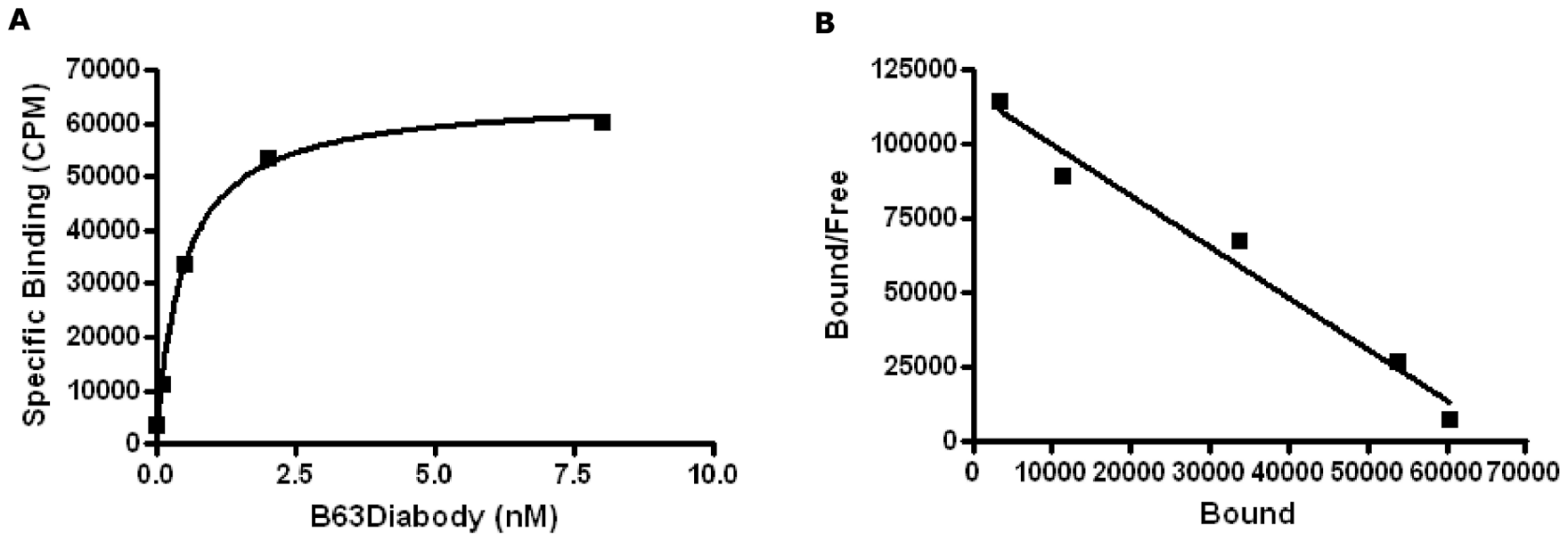
Labeling with ^99m^Tc did not affect B6.3 diabody binding to α_v_β_6_. A) Saturation Binding experiment showed concentration-dependent binding of ^99m^Tc-labeled diabody to A375Pβ6 cells. Non-specific binding, including 25 µg of unlabeled diabody was subtracted from each data point. KD obtained was 4.88±0.32×10^−8^ M and BMax was 2.3±0.039×10^5^ receptors/cell (325±5.53 pM/8.5×10^5^ cells). B) Scatchard presentation of the data. Each experiment was carried out in duplicate.

In order to determine the efficacy of the diabody for α_v_β_6_ targeting *in vivo*, B6.3 diabody was labeled with ^99m^Tc. This radionuclide was chosen as it has been described previously to be appropriate for use with internalizing antibody fragments [28] and the chemistry for conjugation to the hexahistidine tag is commercially available using the IsoLink^Tm^ kit. After labelling, the resulting ^99m^Tc-labeled diabody had a specific activity of 2.7MBq/µg and remained a dimer when tested by size-exclusion chromatography (data not shown). Strength and specificity of interaction of ^99m^Tc-labeled diabody with purified α_v_β_6_ protein was evaluated using a saturation binding experiment which revealed a concentration-dependent increase of ^99m^Tc-B6.3 diabody (Fig. 4A). The specificity of interaction was tested by inhibition of binding by unlabeled diabody. The non-specific binding obtained from the inhibition experiments was subtracted from the total radioactivity to obtain specific binding. When tested on cells, the affinity constant derived from non-linear regression analysis of ^99m^Tc-labeled B6.3 diabody was found to be in the nanomolar range, at 4.88±0.32×10^−8^ M. The maximal number of α_v_β_6_ binding sites (Bmax) was derived to be 2.3±0.039×10^5^ per cell. The Scatchard analysis, which showed a linear correlation (Fig. 4B), was in agreement with a single affinity binding site for the interaction.

### Targeting of B6.3 diabody to α_v_β_6_-expressing tumors *in vivo*

**Figure 5:**
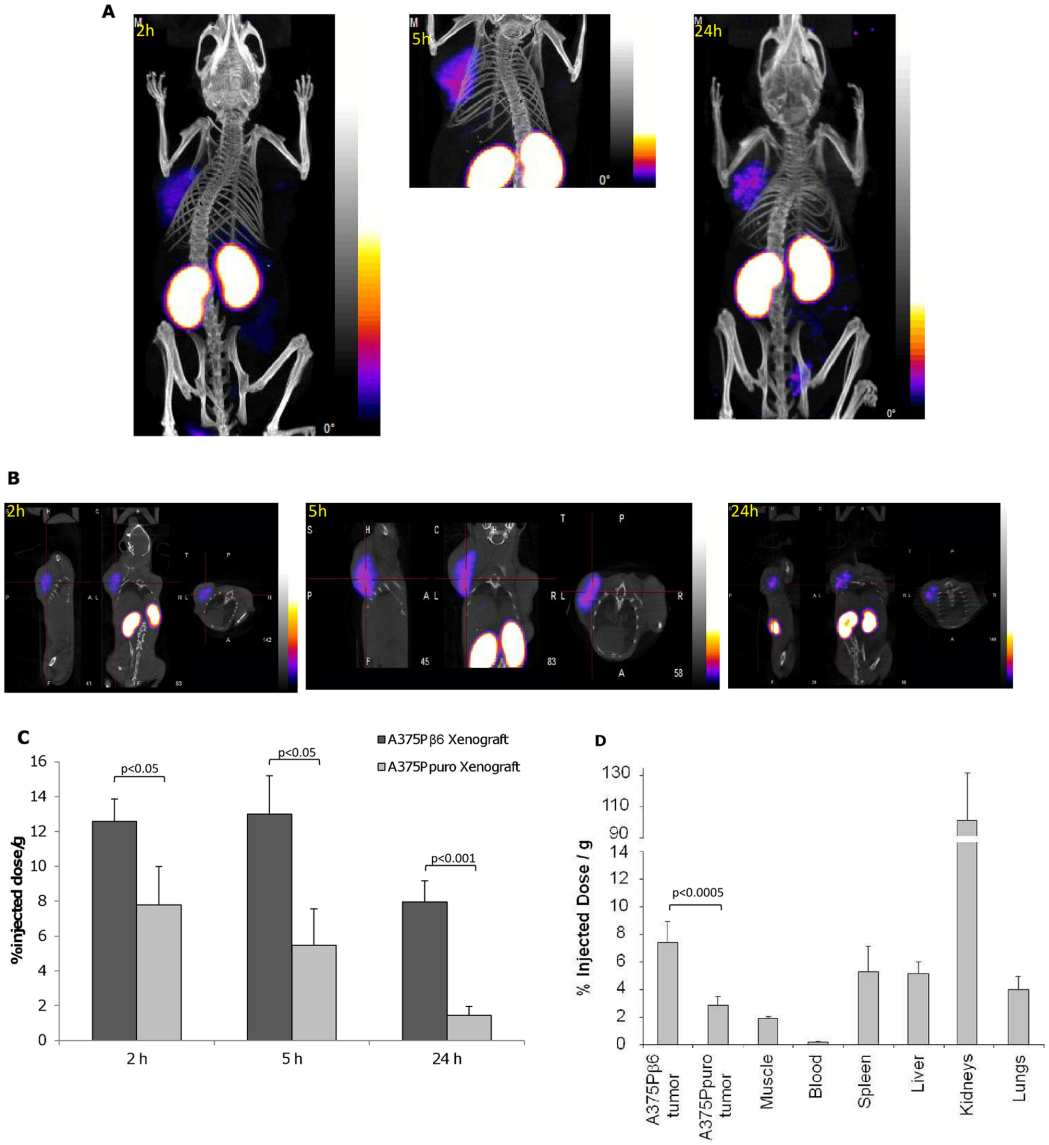
^99m^Tc-labeled B6.3 diabody localised specifically to α_v_β_6_-expressing tumors in vivo. A) A375Pβ6 and A375Ppuro cells were injected subcutaneously on opposite shoulders and ^99m^Tc-labeled B6.3 diabody (approximately 11 µg, 30 MBq) was injected intravenously once tumours had developed. Mice were imaged by SPECT/CT as indicated 2 h, 5 h and 24 h after injection. B) SPECT/CT cross sections of the same mice at 2, 5 and 24 h. C) Percent injected doses of ^99m^Tc-labeled B6.3 diabody in A375Pβ6 and A375Ppuro tumours from three mice, obtained from these images. D) Biodistribution of ^99m^Tc-labeled B6.3 diabody 24 h after injection. Data expressed as % injected dose/g (%ID/g) as mean ± SD for 5 animals. Tumor-to blood ratios at this time point were 40.4 for A375Pβ6 tumors and 15.5 for A375Ppuro tumors. Significance assessed by Student's t-test.

To determine the specificity of the B6.3 diabody *in vivo*, ^99m^Tc-labeled diabody was administered to SCID mice bearing flanking tumors of A375Pβ6 and A375Ppuro cells. Localization of labeled diabody was monitored by whole body cross-section imaging using NanoSPECT/CT. Results of these experiments showed that the α_v_β_6_-expressing A375Pβ6 tumor was detected with the radio-labeled diabody 2h after injection and remained detectable after 5 h and 24 h (Fig. 5A, 5B). Quantification revealed significantly more radioactivity in the α_v_β_6_-expressing tumors when compared to the A375Ppuro tumors at all three time points; the uptake was highest 5 h after injection and considerably reduced after 24 h but remained still clearly detectable (Fig. 5C). The highest normal tissue activity was found in the kidneys, a typical pattern found for radio-metal-labeled compounds due to the excretion of ^99m^Tc-labeled compound by this organ.

Twenty-four hours after injection and imaging, the mice were sacrificed and biodistribution of ^99m^Tc-labeled diabody was determined (Fig. 5D). The data showed that %ID/g obtained in A375Pβ6 tumors was significantly higher (p<0.0005) than that in the A375Ppuro tumors, in agreement with quantification from imaging at this time point. Tumor-to-Blood ratios of 40 were obtained for the α_v_β_6_ positive tumor whereas the α_v_β_6_ negative tumor gave a ratio of 15.5. The kidney had the highest %ID/g of any organ in agreement with the imaging results. Imaging and biodistribution studies showed that ^99m^Tc-labeled diabody targets specifically to α_v_β_6_-expressing tumors *in vivo* and is detectable 24 h after injection with tumor-to-blood ratios suitable for imaging.

## Discussion

This work describes the generation and characterization of a novel diabody that specifically targets the α_v_β_6_ integrin. The diabody was produced as a soluble secreted protein in *P. pastoris,* allowing rapid production using a process readily adaptable to manufacture of clinical grade material [24], [25]. The engineered hexahistidine tag allowed purification and successful labeling with ^99m^Tc, without affecting the nanomolar binding affinity of the diabody on cells *in vitro*.

Our studies also showed that the diabody inhibited adhesion and migration of the α_v_β_6_-transfected melanoma cell line, A375Pβ6 and the pancreatic adenocarcinoma cell line, Capan-1, to LAP. This is the desired function-blocking activity of anti-α_v_β_6_ and has been observed with whole anti-α_v_β_6_ antibodies that block *in vitro* migration of α_v_β_6_–positive Detroit 562 human pharyngeal carcinoma cells and inhibit tumor growth *in vivo* by suppressing TGFβ activation [16], [27]. When interactions via fibronectin, a less specific ligand, were investigated, the diabody was found to inhibit adhesion and migration of Capan-1 cells but not A375Pβ6 cells. This highlights the specificity of B6.3 because A375Pβ6 cells express other fibronectin-binding integrins [17] that would not be blocked by an α_v_β_6_-specific agent. The α_v_β_6_ integrin activates latent-TGFβ first by binding to LAP and then through cortical actin-dependent mechanical forces that causes distortion of the LAP molecule, releasing the TGFβ [29], [30]. Targeting α_v_β_6_ with the B6.3 diabody inhibited this interaction with LAP, resulting in inhibition of Smad2/3 translocation to the nucleus.

The role of α_v_β_6_-dependent TGFβ activation in cancer has been investigated in a number of studies [31], [32], [33], [34], [35], that illustrate both the potential and complexity associated with this target. For example, when α_v_β_6_ was blocked with antibodies in the early stages of disease in a transgenic pancreatic cancer mouse model, this accelerated cancer progression when SMAD4 was functional, but not in SMAD4-null animals [34]. In separate studies α_v_β_6_ promoted cancer growth and liver metastasis through activation of TGFβ [11], [36]. Thus, the functional blockade of α_v_β_6_ has positive therapeutic implications due to the potential inhibition of TGFβ, although TGFβ can also act as a tumour suppressor in normal epithelium and pre-malignant transformed epithelial cells. However, cancer cells often develop mutations that prevent TGFβ-mediated growth inhibition, making TGFβ a strong tumor promoter [32], [33]. Therefore therapeutic antibody blockade of α_v_β_6_ can suppress tumour growth [16], [35] but the molecular phenotype of the tumor must be taken into consideration.

When tested for α_v_β_6_ localization *in vivo*, the radiolabeled diabody showed specific targeting of α_v_β_6_-positive tumours, detectable as early as two hours after injection. Signal was measurable over 24 hours, although intensity was highest five hours after injection. Radiolabeling with ^99m^Tc, using site-specific attachment to the engineered hexahistidine tag, was found to be simple and efficient. Furthermore, use of ^99m^Tc allowed residualization of the signal within the tumor upon internalization of the diabody. These characteristics, combined with the ease of production of B6.3 and its favourable biodistribution *in vivo,* make this diabody an attractive tool for clinical imaging.

The diabody format has not yet been fully exploited as a cancer targeting agent, but it has many attractive features. The bivalency of diabodies conferred by their dimeric structure holds the advantage of higher tumor uptake compared to scFv fragments, resulting in higher signals when used as imaging agents [37]. Also the bigger size of diabodies in relation to scFv increases their circulatory half life, resulting in higher accumulation in the tumor, while achieving better contrast at short time points than bigger engineered fragments such as minibodies [38]. However, despite higher contrast and early imaging, radiometal-labeling of diabodies also results in considerable kidney retention, as shown in our current study and by other groups [22], [37], [38]. This can be problematic if imaging is desirable in close areas. Attachment of polyethylene glycol (PEG) to diabodies has shown to significantly lower kidney retention [22] and improvement of pharmacokinetics [21], resulting in increased circulating time that did not affect the collection of optimal images within 24 hours. The increased circulating time could in fact be advantageous if diabodies are to be used for therapeutic purposes, which required maximum tumor accumulation. In this sense, the internalization of the B6.3 could be clinically useful for delivery of toxic compounds such as the radioisotope, conjugated toxic agents should or small toxic drugs, such as pyrrolobenzodiazepines (PBDs), that are active within target cells.

In summary, the B6.3 diabody described in our study bound specifically to α_v_β_6_ *in vitro* and targeted specifically to α_v_β_6_-expressing tumors *in vivo*. In addition, the diabody retained the biological properties of ligand-mimicking antibodies; it showed internalization upon binding to α_v_β_6_, successfully blocked α_v_β_6_-dependent adhesion and migration to LAP and fibronectin and inhibited smad2/3 nuclear translocation upon treatment with latent TGFβ_1_. Based on its function-blocking activity and specific targeting to α_v_β_6_-positive cells *in vivo*, the B6.3 diabody has potential as an imaging agent or a building block for generation of therapeutics by chemical coupling of small cytotoxic molecules or addition of toxic agents.
